# Self-Assembled Hydrogel Microparticle-Based Tooth-Germ Organoids

**DOI:** 10.3390/bioengineering9050215

**Published:** 2022-05-17

**Authors:** Cemile Kilic Bektas, Weibo Zhang, Yong Mao, Xiaohuan Wu, Joachim Kohn, Pamela C. Yelick

**Affiliations:** 1 Department of Chemistry and Chemical Biology, Rutgers University, 123 Bevier Rd, Piscataway, NJ 08854, USA; cemile.bektas@rutgers.edu (C.K.B.); maoy@chem.rutgers.edu (Y.M.); xiaohuanwu2020@gmail.com (X.W.); kohn@dls.rutgers.edu (J.K.); 2 Division of Craniofacial and Molecular Genetics, Department of Orthodontics, Tufts University School of Dental Medicine, 1 Kneeland Avenue, Boston, MA 02111, USA; weibo.zhang@tufts.edu

**Keywords:** hydrogel microparticles (HMPs), tooth-bud organoids, GelMA microparticles, human dental pulp stem cells (hDPSCs), porcine dental endothelial cells (pDE), self-assembled organoid structures

## Abstract

Here, we describe the characterization of tooth-germ organoids, three-dimensional (3D) constructs cultured in vitro with the potential to develop into living teeth. To date, the methods used to successfully create tooth organoids capable of forming functional teeth have been quite limited. Recently, hydrogel microparticles (HMP) have demonstrated utility in tissue repair and regeneration based on their useful characteristics, including their scaffolding ability, effective cell and drug delivery, their ability to mimic the natural tissue extracellular matrix, and their injectability. These outstanding properties led us to investigate the utility of using HMPs (average diameter: 158 ± 32 µm) derived from methacrylated gelatin (GelMA) (degree of substitution: 100%) to create tooth organoids. The tooth organoids were created by seeding human dental pulp stem cells (hDPSCs) and porcine dental epithelial cells (pDE) onto the HMPs, which provided an extensive surface area for the cells to effectively attach and proliferate. Interestingly, the cell-seeded HMPs cultured on low-attachment tissue culture plates with gentle rocking self-assembled into organoids, within which the cells maintained their viability and morphology throughout the incubation period. The self-assembled organoids reached a volume of ~50 mm^3^ within two weeks of the in vitro tissue culture. The co-cultured hDPSC-HMP and pDE-HMP structures effectively attached to each other without any externally applied forces. The presence of polarized, differentiated dental cells in these composite tooth-bud organoids demonstrated the potential of self-assembled dental cell HMPs to form tooth-bud organoid-like structures for potential applications in tooth regeneration strategies.

## 1. Introduction

Natural tooth development is the product of carefully orchestrated reciprocal interactions between the dental epithelium (DE) and the dental mesenchyme (DM) [1]. Facilitating proper DE–DM cell crosstalk is therefore essential to bioengineer constructs for functional whole-tooth regeneration. To date, tooth germ organoids have been created from easily obtainable mouse embryonic tooth-bud tissues and cells [2,3,4]. By contrast, human embryonic dental tissues are extremely difficult. if not impossible, to obtain. Using mouse embryonic DE–DM cells, efficient crosstalk was accomplished by combining DE and DM cell pellets directly within a collagen drop [5,6] that was subsequently cultured in semi-solid agar media [7], or by using a Trowell in vitro culture device [8]. These studies showed that the size of the regenerated teeth correlated with the size of the cell pellets within the collagen drop. Although embryonic mouse-tooth-derived regenerated teeth were comparable in size to natural mouse teeth, they were much smaller than human teeth. A similar approach using human gingival and dental cells resulted in the formation of multiple small teeth of aberrant shapes and sizes [9]. Together, these results revealed that alternative methods are needed to successfully create in vitro tooth organoids for human tooth regeneration.

Hydrogels are 3D hydrophilic polymeric networks that can retain water of up to a thousand times their dry weight [10]. Due to their significant similarity to the native tissue extracellular matrix (ECM), hydrogels have been widely used in many applications, including tissue engineering [11], drug and gene delivery [12], cell therapy [13], pharmaceuticals [14], and biosensors [15]. Hydrogels must meet certain criteria to be used in successful tissue engineering applications, including biocompatibility, biodegradability, and appropriate mechanical strength. Naturally derived polymers are often considered for tissue engineering applications due to their excellent biocompatibility [16]. Gelatin, the denatured form of collagen, has been used in a variety of tissue engineering applications due to its low immunogenicity and highly biocompatible nature [17]. Gelatin methacrylate (GelMA) is a gelatin derivative synthesized by combining gelatin and methacrylic anhydride (MAA) [11,18]. GelMA hydrogels typically contain ~5% MAA, and, therefore, retain a large number of natural cell-binding motifs, including arginine–glycine–aspartic acid (RGD), which promotes cell adhesion and matrix metalloproteinase (MMP) secretion, both of which are crucial for ECM remodeling.

GelMA has been identified as a promising hydrogel for dental tissue regeneration due to its excellent biodegradability, bioactivity, and tailorability with respect to porosity, mechanical strength, and degradation rates. GelMA was shown to support human dental pulp stem cell (hDPSC) growth and differentiation when tested in bulk [19,20] or in 3D-printed forms [21]. Our previous study showed the possibility of regenerating full-sized human tooth buds (TB) using GelMA-encapsulated dental cells [22,23]. However, the viability of encapsulated cells and effective cell–cell signaling within GelMA, has remained a challenge.

Although cell encapsulation in hydrogels is a promising approach in general, when cells are encapsulated in large volumes, they behave as bulk hydrogels, thus limiting the accessibility of nutrients and oxygen to encapsulated cells, resulting in reduced cell viability and functionality [24]. Hydrogel microparticles (HMPs) (~1–1000 µm in size) provide a promising solution to certain limitations imposed by encapsulation by providing high surface areas for cell attachment and allowing for direct cell–cell interactions. Furthermore, cells, drugs, and growth factors can be encapsulated in HMPs of various sizes and shapes using fabrication techniques, including microfluidics, batch emulsion, and mechanical fragmentation [24]. Although HMPs have been widely used in tissue engineering [25,26] and drug delivery research [27,28], their use in dental tissue regeneration has not been fully explored. To date, several polymeric and hydrogel-based microspheres have been employed in the dental field. Although synthetic polymers, such as poly(L-lactic acid) [29], poly(L-lactic acid)-block-poly(L-lysine) [30], and GelMA [31], have been characterized over the past few years, and even decades, concerns were raised regarding their slow degradation rates and the potential for acidic degradation products to induce inflammation [32,33]. Recently, several studies have reported the use of hDPSC-encapsulated HMPs for endodontic tissue regeneration, including RGD (arginine–glycine–aspartic acid)-alginate-based [34] and GelMA-based HMPs [31]. However, the RGD-alginate-based HMPs showed slow degradation rates and poor cell spreading, while the GelMA-based HMPs exhibited limited cell density due to the hollow shapes of the HMPs.

To overcome the various limitations of these encapsulation techniques, in this study, we seeded cells directly onto GelMA microparticles. Because these cell-seeded HMPs self-assembled into tissue-like organoids, the size of the structures was rendered scalable by increasing the number of MPs and cells. This approach provides the opportunity to generate dental-cell-seeded HMP-derived organoids exhibiting excellent viability, and that are readily adaptable to match a given tooth bud geometry. Importantly, our approach provides an easy, highly adaptable model for fabricating a variety of cell-seeded HMP organoid-like structures that mimic native tissue structures.

## 2. Materials and Methods

### 2.1. Preparation of Methacrylated Gelatin

Gelatin Type A from porcine skin (300 bloom, Sigma Aldrich, St. Louis, MO, USA) was dissolved at 10% (*w*/*v*) in 100 mL of 0.25 M carbonate-bicarbonate (CB) (Sigma Aldrich) buffer at 55 °C. The pH of the solution was adjusted to 9.4, and 0.938 mL methacrylic anhydride (Sigma Aldrich) was added to the solution slowly under magnetic stirring at 500 rpm to achieve a targeted 100% degree of substitution (DS). The reaction was continued for 1 h at 55 °C and stopped by decreasing the pH of the solution to 7.4. After filtration, the solution was dialyzed for 2 days and lyophilized to obtain a white solid product. The yield in each of the three batches was 90%.

#### 2.1.1. H-NMR Analysis

The degree of substitution (DS) was analyzed from ^1^H-NMR spectra of gelatin and GelMA to quantify the methacrylate groups in GelMA using a Bruker DPX 500 spectrometer. Briefly, 30 mg of GelMA and gelatin were each dissolved in 1 mL of deuterium oxide (Acros Organics, Morris Plain, NJ, USA) at 40 °C and H-NMR spectra were collected at 40 °C. After phase and baseline corrections, the chemical shift scale was adjusted to the phenylalanine signal (6.9–7.5 ppm). The NMR spectra was also normalized to the phenylalanine signal, which was proportional with the concentration of the gelatin. DS was calculated by integrating the areas of lysine methylene signals (2.8–2.95 ppm) of GelMA and gelatin according to the following equation:

(1) $$\mathrm{DS}\;\left(\%\right)=\left(1-\frac{\mathrm{Peak}\;\mathrm{area}\;\left(\mathrm{lysine}\;\mathrm{methylene}\;\mathrm{of}\;\mathrm{GelMA}\right)}{\mathrm{Peak}\;\mathrm{area}\;\left(\mathrm{lysine}\;\mathrm{methylene}\;\mathrm{of}\;\mathrm{non}-\mathrm{modified}\;\mathrm{gelatin}\right)}\right)\times 100$$

#### 2.1.2. ATR-FTIR

FTIR spectra of gelatin, GelMA, and GelMA-HMPs were obtained with Thermo Scientific FTIR spectrometer (Model Nicolet iS10, Madison, WI, USA) in the range between 4000 and 400 cm^−2^.

#### 2.1.3. Preparation of GelMA Microparticles

**Figure 1:**
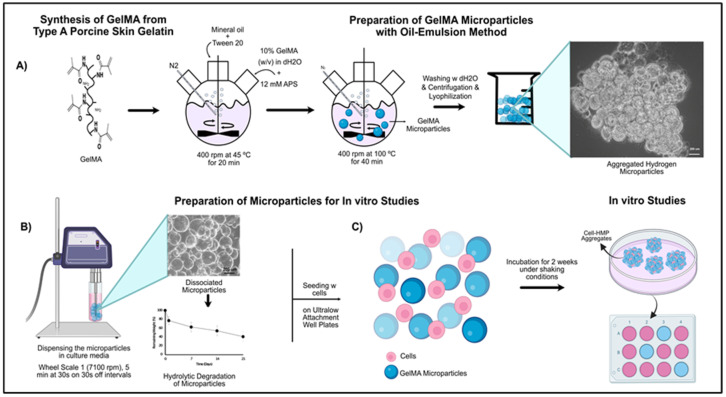
Experimental approach: (**A**) GelMA HMPs were synthesized from type A porcine skin gelatin using oil–water emulsion method followed by thermal crosslinking at 100 °C followed by lyophilization. (**B**) Aggregated HMPs were dissociated via sonication. (**C**) Cell-seeded HMPs were cultured on ultralow attachment plates for 2 weeks with constant shaking.

Lyophilized GelMA was fully dissolved in 5 mL diH_2_O (10%, *w*/*v*) at 37 °C at RT. Next, 75 mL of mineral oil (Sigma Aldrich) was heated to 37 °C in a two-neck 250-mL round-bottomed flask prior to the addition of 1 mL of Tween 20 (Thermo Fisher Scientific, Waltham, MA, USA) with stirring at 400 rpm for 5 min. The 10% GelMA solution was mixed with 14 mg (12.5 mM) ammonium persulfate (APS) (Sigma Aldrich) and added dropwise to the oil phase. The oil–water emulsions were stirred at 400 rpm 5 min, then N_2_ gas was bubbled through the emulsion for 20 min at 45 °C to purge oxygen. Under constant stirring, the temperature was increased to 100 °C, and thermal cross-linking reaction was allowed to occur for 40 min. The mixture was then centrifuged at 2500 rpm at 4 °C to harvest the microparticles. The pellet was washed three times with dH_2_O, followed by centrifugation to remove any oil residue. Finally, the pellet was freeze-dried and stored at 4 °C until further use (Figure 1A). Microparticles (MPs) were monitored under optical microscopy (Zeiss Axio Observer D1, Jena, Germany) before and after lyophilization in diH_2_O. The average particle size was analyzed using NIH Image J analysis software.

#### 2.1.4. In Situ Degradation of GelMA Microparticles

GelMA hydrogel microparticles (HMPs) (6–8 mg, $W_{o}$) were incubated in PBS containing 0.05% (sodium azide (Sigma Aldrich) at 37 °C under shaking conditions. At predetermined time points (Days 1, 7, 14 and 21) samples were centrifuged and washed three times with sterile water (WFI) (Gibco, Gaithersburg, MD, USA), and the resulting pellet was freeze-dried and weighed ($W_{d}$). Remaining weight was calculated according to following equation:

(2) $$Remaining\;Weight=\frac{W_{d}}{W_{o}}\times 100$$

### 2.2. In Vitro Biocompatibility Tests

hDPSCs. hDPSCs were isolated at Tufts University, as previously described [35]. Briefly, dental pulp was isolated from human teeth extracted at the Tufts University School of Dental Medicine for clinically relevant reasons. Dental pulps were minced into small pieces, digested using 0.3 mg/mL collagenase type I (Sigma-Aldrich) and 0.4 mg/mL dispase (Boehringer Mannheim, Indianapolis, IN, USA), and cultured in DMEM/F12 media with 10% fetal bovine serum (FBS, Gibco, MD, USA), 1% GlutaMAX (Gibco), 50 µg/mL ascorbic acid (Sigma Aldrich), and 1% penicillin/streptomycin/amphotericin (PSA) (Gibco), with media changes every 2~3 days. The human DPSCs were expanded up to passage 3 and then cryopreserved until use. Prior to use in experiments, hDPSCs were thawed and expanded using the same methods.

Human Dermal Fibroblast (hDF) Cell Culture. HDFs (passage 8) were initially used as a control cell type to test the biocompatibility of GelMA HMPs prior to using hDPSCs. Cryopreserved hDFs were thawed and expanded in growth media consisting of DMEM high glucose (Gibco) supplemented by 10% FBS and 25 µg/mL gentamicin (Sigma Aldrich).

#### 2.2.1. Seeding of hDPSCs or HDFs onto GelMA Microparticles

GelMA HMPs were sterilized under UV for 1 h and suspended in hDPSC growth media (2 mg/1 mL of growth media). The GelMA HMP solution was then sonicated under sterile conditions for 5 min (30 s on and 30 s off) using an Ika T10 basic Ultra-Turrax Dispenser (IKA Works, Inc., Wilmington, NC, USA) at wheel scale 1 (7100 rpm) (Figure 1B). Next, 1 mL of GelMA HMP solution was pipetted into each well of 24-well ultralow attachment plates.

In the meantime, hDPSCs or HDF cells were prepared as follows. hDPSCs/HDF cells were seeded in T75 flasks, grown to 90% confluency, and harvested using trypsin (Gibco). Next, a 250 μL (2.5 × 10^5^ cells/well) cell suspension was seeded onto replicate (3) HMPs, and replicate (3) HMPs alone and cells alone served as controls. The plates were incubated on a rocker (Rocker II, Koekel Scientific, USA) in a continuous rocking mode at 37 °C, 5% CO_2_ cell culture incubator for 2 weeks (Figure 1C). Media was changed every other day.

#### 2.2.2. Alamar Blue Cell Viability Assay

The viability and metabolic activity of cultured hDPSC or hDF cells was assessed using AlamarBlue^®^ assay (Carlsbad, CA, Bio-Rad, Philadelphia, PA, USA). Replicate samples (*n* = 3) were incubated in 10% (*v*/*v* in respective media without FBS) AlamarBlue^®^ solution for 30 min. Reduction (%) in the dye was calculated according to the manufacturer’s instructions, using fluorescence at excitation of 540 nm and emission of 590 nm at 100% gain using fluorescence plate reader (TECAN Spark, TECAN, Morrisville, NC, USA). Fluorescence of HMPs alone served as control and was subtracted from fluorescence reading of cell-containing HMP samples.

#### 2.2.3. Live-Dead Cell Viability Assay

The viability of the cells (%) was determined using Live-Dead cell viability assay (Thermo Fisher Scientific) according to the manufacturer’s instructions. Briefly, cells were double-stained with Calcein AM (2 µm in PBS) and ethidium homodimer-1 (4 µm in PBS) for 30 min at RT, washed with PBS and examined using a Zeiss LSM 710 (Oberkochen, Germany) confocal laser scanning electron microscope. HMP-cell tissue clusters were cut half using a surgical blade to observe them in cross-section.

#### 2.2.4. FITC Phalloidin and Hoechst Staining

Tissue-like HMP human cell clusters were fixed for 30 min in 4% paraformaldehyde (PFA) at RT, washed twice and incubated in sucrose (20% solution) O/N. Next, samples were embedded in optical cutting temperature compound (OCT) (Miles Scientific, Naperville, IL, USA) and frozen at −80 °C. Samples were serially sectioned (6 µm) using a Leica cryostat (Nussloch, Germany) and stained with FITC-labeled Phalloidin (Hyclone, Thermo scientific) and Hoechst (AnasPec, Fremont, CA, USA). Briefly, sections were treated with 0.1% Triton X (Fisher Scientific) (*v*/*v*, in PBS) for 5 min, washed twice with PBS and incubated with 1% BSA for 1 h at 37 °C to prevent nonspecific binding of the dyes. Next, sections were stained with FITC Phalloidin (1:50 in 0.1% BSA) for 1 h at 37 °C. After washing twice with PBS, sections were stained with Hoechst (1:500 in PBS) for 30 min at RT. Samples were washed twice with PBS and examined under a fluorescence microscope (Zeiss Axiophot Imager, Zeiss, Berlin, Germany).

#### 2.2.5. Tooth Bud Organoid Recombination Testing

Tooth-bud organoids were constructed by combining DE- and DM-HMP cell clusters. The DM cell source was the hDPSCs mentioned above, while the DE cell source was harvested from enamel organ (EO) tissues of 5 1/2-month-old porcine tooth buds (pDE). The pDE cells were cultured in LHC-8 medium (Gibco), containing 10% FBS, 0.5 µg/mL Epinephrine (Sigma), and 1% PSA as described previously [36]. The hDPSC and pDE cells were separately seeded onto GelMA HMPs, as described above, at a density of 2.5 × 10^5^ cells/2 mg HMP/culture, in ultralow attachment 24-well plates. After one week of culture, pDE-HMP and hDPSCs-HMP constructs were combined and cultured with rocking in ultralow attachment 24-well plates for an additional two weeks in DE:DM (1:1) media. Uncombined pDE-HMP, hDPSCs-HMP, hDPSC or pDE cells without HMPs, and HMPs alone were cultured as controls (see Supplemental Figure S1). After harvest, Live-Dead assay was performed using a LIVE/DEAD ^®^ Viability/Cytotoxicity Kit. The samples were then fixed, processed, and embedded in optimal cutting temperature (OCT) for cryosection. The 10-micrometer cryosections were then stained with hematoxylin and eosin (H&E).

## 3. Results

### 3.1. ^1^H-NMR Analysis of GelMA

**Figure 2:**
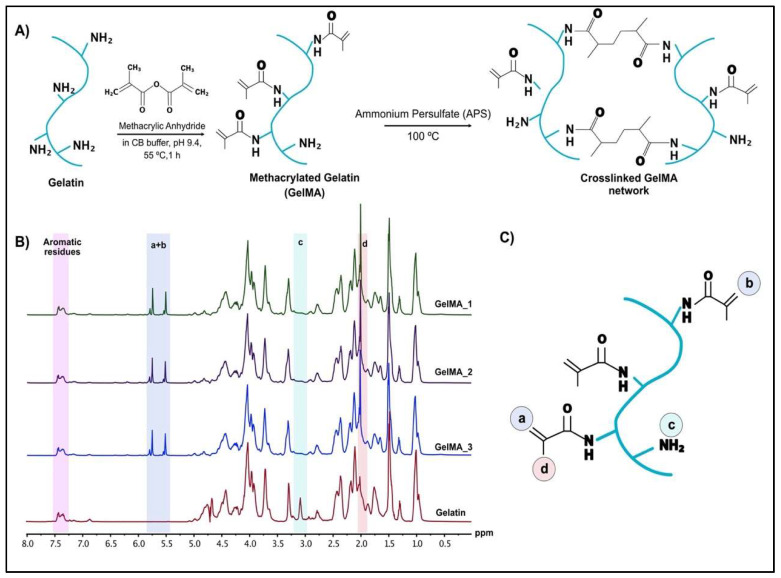
Methacrylated gelatin synthesis and 1H-NMR spectra of gelatin and GelMA: (**A**) Reaction scheme of gelatin with methacrylic anhydride to form GelMA. (**B**) ^1^H-NMR spectra of gelatin and three batches of GelMA (GelMA_1, GelMA_2 and GelMA_3) in DH_2_O. (**C**) Specific protons indicated on the 1H-NMR spectra are highlighted on the GelMA structure where: (a) and (b) indicate acrylic protons of methacrylamide groups in lysine and hydroxylysine residues, respectively; (c) indicates methylene protons of unreacted lysine; and (d) indicates methyl protons of methacrylamide groups.

The methacrylated gelatin was prepared from gelatin and methacrylic anhydride using a recently reported one-pot GelMA synthesis protocol [37] (Figure 2A). The batch-to-batch consistency and degree of controllability were validated when the reaction was carried out in CB buffer (pH 9.4) at 55 °C for 1 h. A 100% degree of substitution (DS) was targeted and calculated from the NMR spectra of gelatin and GelMA using MestreNova NMR analysis program (Figure 2B). The spectra were normalized to phenylalanine signal (6.9–7.5 ppm), which was related to the concentration of the gelatin. The decreased lysine methylene signal (2.8–2.97 ppm) (Figure 2B,C) and the ratio of the integrated areas were used to calculate the DS of the GelMA. A 100% DS was achieved with a 90% yield in three different batches, which is consistent with the results reported in other studies [37]. The successful methacrylate modification of gelatin was also verified by FTIR spectra of gelatin, GelMA and GelMA HMPs [38] (Figure S2).

### 3.2. Characterization of Human Cell HMP Tissue Constructs

#### 3.2.1. Characterization of Fabricated HMPs

The HMPs were relatively uniform, with an average size of 158 ± 32 µm (min: 123 µm, max: 193 µm) in diH_2_O. The scaled-up production of the HMPs using an oil–water emulsion method resulted in yields as high as 90%, which enabled us to use the same batch of HMPs throughout the study. The lyophilized HMP samples were stored at 4 °C for long-term use without any function or form change as validated using light microscopy prior to each in vitro study. The lyophilization resulted in HMP aggregation, which decreased the surface-to-volume ratio and cell attachment surface area. The aggregation problem was solved by dispensing the HMPs into individual tubes, followed by dissociation via rotation at low speed (wheel scale 1, 7100 rpm) for 5 min (at intervals of 30 s on and 30 s off) prior to in vitro culture studies. Increasing the speed and/or dispersion time resulted in the HMPs’ fragmentation into irregular shapes (Figure S3).

**Figure 3:**
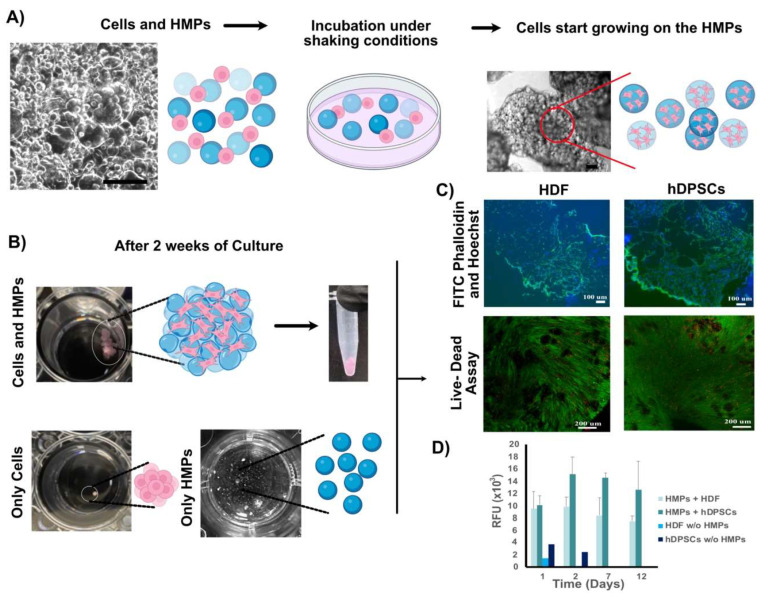
In vitro biocompatibility tests of self-assembled cell-HMP aggregates: (**A**) hDF cells or hDPSCs seeded together with GelMA hydrogel microparticles (GelMA HMPs) and incubated in ultralow attachment plates with rocking formed tissue-like constructs by Day 1. Scale bars in A: 200 µm. (**B**) After 2 weeks of culture cell-HMPs formed tissue-like aggregates that were easy to handle. Cells without HMPs formed ten times smaller clusters, and HMPs alone did not adhere together, but rather remained distributed throughout the media. (**C**) Cryosections (5 µm in thickness) of tissue-like clusters stained with FITC-Phalloidin (upper panel) and with Live-Dead dyes (lower panel). Scale bars: 100 µm (top) and 200 µm (bottom). (**D**) Cell viability in the presence or absence of HMPs was measured using an Alamar Blue viability assay over 12 days (data shown are mean ± SD, *n* = 3) Abbreviations: HDF, human dermal fibroblasts; hDPSCs, human dental pulp stem cells; HMPs, hydrogel microparticles.

The biocompatibility of the HMP cultured with HDF and hDPSCs cultured in ultralow-attachment 24-well plates was assessed as follows. The behavior of the cultured cells and HMPs was monitored under optical microscopy each day for 2 weeks. Cell-HMP constructs ranged in size from 50 µm to 150 µm in diameter on Day 0 and were easily distinguishable using light microscopy (Figure 3A). However, starting from Day 1, the cells and HMPs began to self-assemble as the cells attached and proliferated on the HMPs, and behaved like adhesivse, holding the HMPs together (Figure 3A, top-right image). By contrast, the HMPs cultured without cells remained apart during the entire culture period, and the cells cultured without HMPs formed small clusters and lost their viability after 1 week of incubation (Figure 3B). Self-assembled cell–HMP aggregate clusters (~50 mm^3^ in size) formed after 4 days of culture and were stable enough to manipulate using forceps without damage (Figure 3B). In this study, similar-sized clusters were obtained using HDFs and hDPSCs, suggesting that cell-HMP self-assembled aggregates generated from different cell types could be combined to obtain multilayered composite structures.

To evaluate cell density and morphology throughout the 3D constructs, cell-HMP aggregates were cut in half, and cross-sectional images were obtained using light microscopy. These analyses showed highly dense cell-to-cell interactions surrounding HMPs (Figure 3C). Although some cell death was observed via the Live-Dead assay of the cross-sectioned constructs, most of the cells were alive and metabolically active. The cells cultured without HMPs formed very small clusters (Figure 3B) and AlamarBlue^®^ assay indicated reduced cell viability after 1 week (Figure 3D).

The self-assembled cell-HMP aggregates were mechanically robust, allowing easy handling, and maintained their integrity during the cryosectioning. The FITC-Phalloidin and Hoechst staining of the actin filaments and nuclei, respectively, were used to analyze cell morphology in sectioned self-assembled cell-HMP aggregates. While strong phalloidin staining was observed along the periphery of the aggregates, indicating the formation of dense stress fibers and increased spreading of the cells along the periphery of the aggregates (Figure 3C upper panels), viable cells were uniformly distributed throughout the aggregates, including in the center (Figure 3C lower panels). Both the hDPSC and the HDF cell type showed excellent biocompatibility with the HMPs (Figure 3C).

#### 3.2.2. Tooth-Bud Organoid Recombination Test

**Figure 4:**
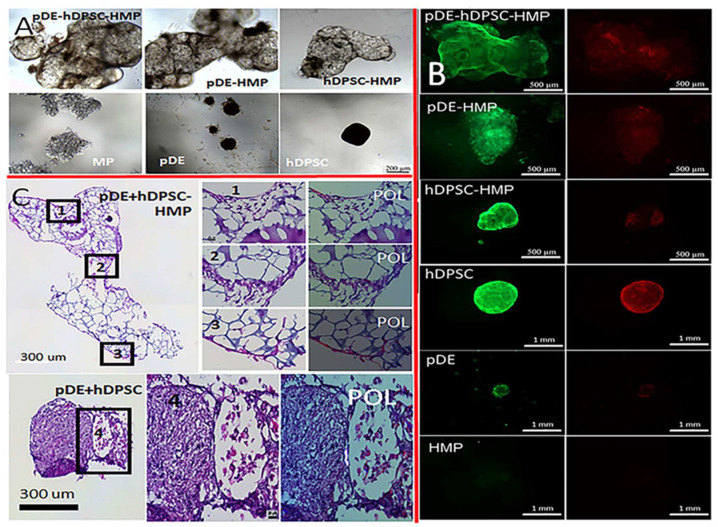
Tooth-bud organoids after 1-week in vitro culture: (**A**) Bright field image of co-cultured pDE and hDPSC cell clusters shows that clusters cultured with HMPs appear larger and better-organized than those without HMPs. (**B**) Live-Dead assay showed higher viability for cells cultured with HMPs. Green fluorescence indicates live cells, and red fluorescence indicates dead cells. (**C**) Brightfield and polarized light microscopy (POL) of H&E-stained co-cultured constructs showing that pDE cells displayed a typical clustered morphology (1) while hDPSCs exhibited typical spindle shaped morphology (3). Clear cell–cell contact between polarized cell types was observed in constructs with HMP (2), but not in constructs without HMP, although both types of cell were present in co-cultured constructs (4). Scale bar = 200 µm (**A**), 500 µm ((**B**), with HMP), 1 mm ((**B**), cell alone), 300 µm (**C**), 20 µm (Panels 1–4, 1′–4′). Analyses of 2-week-cultured constructs are shown in Supplementary Figures S4 and S5.

The cultured pDE-HMP and hDPSC-HMP clusters appeared distinct when viewed under light microscopy (Figure 3 and Figure 4), in that the self-assembled pDE-HMPs showed multiple, smaller clusters compared to the one larger cluster observed in the cultured hDPSC-HMPs. The hDPSCs cultured alone without any HMPs formed a single small cluster, while the pDE cells alone without HMPs formed even smaller, multiple clusters. The HMPs without cells remained evenly distributed and unclustered (Figure 4). When the pDE-HMP and hDPSC-HMPs were combined in culture, after one day, a single large, self-assembled-pDE-hDPSC-HMP cluster formed in each well, indicating that the cell-seeded HMPs adhered together to form larger pDE-HMP and hDPSC-HMP clusters. The Live-Dead assay showed higher cell viability in the cell-HMP constructs compared to the cells cultured alone, both before and after recombination (Figure 4B). Higher numbers of dead cells with stronger red staining were observed in 2-week cultured constructs compared to 1-week cultured constructs, with or without HMPs. H&E staining showed the successful recombination of pDE-HMP and hDPSC-HMP clusters after 1 week co-culture (Figure 4C). Combined pDE and hDPSC cells without HMPs also formed one cluster, which was significantly smaller in size and of higher cell density than the cell-HMP clusters (Figures S4 and S5). Evidence for distinct and clear DE–DM cell contacts was detectable in the combined pDE and hDPSC cell cultures with or without HMPs, while cell polarization indicative of dental cell differentiation was only observed in the cells cultured with HMPs (Figure 4C-2, 2′) versus (Figure 4C-4, 4′).

## 4. Discussion

Successful organ regeneration can be achieved via the reliable generation of tissue-like organoids that display cellular interactions, architectures, and functionalities similar to those of in vivo tissues and organs. The ability to mimic the spatio-temporal interactions that occur between DE and DM tissues during natural tooth development is essential for whole-tooth regeneration. The main purpose of this study was to validate whether co-cultured dental-cell-seeded HMP constructs could be used to create a composite dental tissue organoid exhibiting a functional DE–DM tissue interface. Tooth-bud-like organoids with DE–DM tissue interactions mimicking those of native tooth-buds have potential utility for whole-tooth regeneration.

Natural hydrogel biomaterials have been extensively studied for tissue engineering applications [39,40]. Among them, gelatin is one of the most widely used hydrogel biomaterials due to its low immunogenicity, retention of motifs for cell binding, and ability to be degraded by MMPs [17]. GelMA is one of the most popular derivatives of gelatin due to its tunable chemical structure, biocompatibility and biodegradability, and suitability for the attachment and growth of many cell types [11,41]. In this study, GelMA was fabricated using the recently published one-pot GelMA synthesis protocol [37], which offers advantages over other fabrication methods, including a short reaction and purification time, batch-to-batch consistency, and a high degree of controllability [37,42]. We achieved a 100% degree of substitution (DS) in this study by using this method.

The GelMA hydrogel precursor was then used to produce GelMA HMPs using batch emulsion techniques, through which the dispersity and size of the HMPs can easily be modulated by varying the stirring speed and duration [43]. HMPs obtained in this study were then incubated with three different cell types, hDPSCs, HDFs, and pDE cells, providing a highly suitable environment for cell attachment and growth. Owing to their high water content, HMPs resemble native ECM and can be tailored to include proteins or attached cells. Yang et. al. studied the endodontic regeneration potential of hDPSC-laden GelMA microspheres and reported the generation of abundant blood vessels in GelMA microspheres compared to bulk GelMA hydrogels [31]. Khayat et al. demonstrated that hDPSCs and HUVECs encapsulated in GelMA supported robust vascularization in a subcutaneous implanted-tooth-slice assay [19]. In a similar study, Zhang et al. reported co-encapsulating hDPSCs and vascular endothelial growth factor (VEGF) in RGD-alginate/laponite microspheres for endodontic regeneration and showed new micro-vessel generation [34]. Although cell encapsulation into microspheres is a promising approach, it has inherent limitations, including reduced cell–cell contact, poor cell spreading, and low-throughput fabrication. By contrast, in this study, the cells attached and proliferated on the HMPs, and, even more strikingly, co-cultured HMPs and cells formed self-assembled aggregates in as little as four days. The self-assembled aggregates were mechanically stable, supported high cell viability and expected cell morphology, and grew as large as 50 mm^3^ in diameter over a 2-week in vitro culture period. The aggregation of GelMA microspheres and encapsulated cells was reported previously [31]; however, the volumes of the aggregated clusters were much smaller than those observed in this study (1.8 mm^3^ vs. 50 mm^3^), even after 6 weeks of in vitro culture. Here, we demonstrate that the cluster volumes obtained in this study can be tailored by scaling up the amounts of cells and HMPs used, and the self-assembled Cell-HMP aggregates can subsequently be combined to form larger organoids. Therefore, the HMPs utilized in this study have several significant advantages over cell encapsulation in bulk hydrogels, as follows. (1) HMPs offer a biomimetic 3D ECM microenvironment that allows rapid oxygen and nutrient diffusion and effective cell–cell interactions compared to bulk hydrogels. In addition, waste products are not accumulated in the constructs, which could otherwise negatively affect the cell viability, proliferation, differentiation, and product quality [44]. (2) The high surface-to-volume ratio of HMPs provides an excellent platform for cell attachment and tissue growth. (3) The high cell viability and density supported by HMPs promotes efficient cell–cell signaling and tissue regeneration to a much greater extent than HMP-encapsulated cells. (4) HMPs provide structural stability to cell-seeded organoids. (5) Controlled scale-up and the desired tissue complexity can easily be achieved by combining self-assembled HMP aggregates containing a variety of cell types. (6) The HMPs showed enhanced biocompatibility compared to bulk hydrogels, which typically require toxic chemical crosslinkers or prolonged irradiation times for in situ gel formation [45].

To date, our laboratory has used a variety of approaches for tooth regeneration. We showed that pDE cells and hDPSCs seeded onto electrospun PLGA/PCL nHA fibrous scaffolds formed multiple, small tooth-like structures throughout the construct, demonstrating effective DE–DM cell interactions for tooth regeneration, although the slow scaffold degradation rate may have contributed to disordered dental tissue formation [46]. We also demonstrated that the decellularized porcine tooth-bud scaffolds (dTBs) supported the formation of mature tooth-like structures [47]. Recently, we created bioengineered tooth-bud constructs exhibiting many features of natural tooth buds by combining GelMA-encapsulated pDE cells/human umbilical-vein endothelial-cell (HUVEC) bilayers with GelMA-encapsulated hDPSCs/HUVEC bilayers [22]. The analyses of these constructs cultured in vitro or implanted subcutaneously in vivo showed an even distribution of cells within the GelMA bilayers, but little evidence for direct DE and DM cell contact at the bilayer interface, and, subsequently, limited DE or DM cell polarization, indicative of dental cell differentiation. These results indicated the need to identify alternative approaches that would facilitate DE- and DM-cell interactions for effective tooth regeneration.

Based on the many advantages of the self-assembled cell-seeded HMP aggregates, we tested their utility for tooth-bud organoid formation by combining pDE- and hDPSC-HMP constructs. Our results showed that the direct DE–DM cell–cell contacts achieved by the self-assembled aggregates resulted in clearly visible polarized, differentiated dental cells at the interface of the DE-HMP and DM-HMP constructs. Direct DE–DM cell contact was also observed in the constructs without HMPs, but no polarized cells were observed, which indicated that HMPs benefit the vitality of functional DE–DM tissue interactions. Together, these observations demonstrated that GelMA HMPs with high biocompatibility and surface area facilitated dental cell attachment and proliferation, and controlled HMP degradation in response to seeded cells and DE–DM cell–cell signaling, all of which benefitted tooth organoid formation.

## 5. Conclusions

Three-dimensional (3D) organoids, which are self-organized cell structures, can mimic the dynamic growth and differentiation observed in natural organs. Organoids generated using nanomaterials can closely approximate in vivo tissue-like behavior, and provide realistic models for investigating the mechanisms regulating tissue development and disease. In this study, we described a novel approach to generating scalable tooth organoids using dental-cell-seeded GelMA HMPs. Our results showed that both the pDE cells and the pDPSCs readily attached and proliferated on the HMPs to form aggregates exhibiting robust mechanical properties, which could subsequently self-assemble under gently rocking conditions into dental tissue organoid-like structures. Furthermore, the pDE-HMP and hDPSC-HMP aggregates were combined to form tooth-bud-like organoids, which supported the formation of a DE–DM cell-layer interface that facilitated beneficial DE–DM cell crosstalk and dental cell differentiation. This study introduces an advanced method for tooth-tissue engineering that can also be used to regenerate other types of organ using a similar approach. Future studies, including comparative studies with commonly applied hydrogel microparticles (e.g., PEG-based), more detailed analyses of DE–DM cell–cell signaling in bioengineered HMP-derived toot- bud organoids, the demonstration of the scaled-up fabrication of dental cell HMP organoids, and in vivo analyses of implanted constructs will help to further define and validate this approach.

## Data Availability

Not applicable.

## Supplementary Materials

The following supporting information can be downloaded at: https://www.mdpi.com/article/10.3390/bioengineering9050215/s1, Figure S1: Flowchart of Tooth Bud Organoid Combination Testing; Figure S2: FTIR-ATR spectra. GelMA and GelMA hydrogel MPs exhibited the following peaks; Figure S3: Effect of dispersion speed and duration on the microparticles.; Figure S4: Two week in vitro cultured Tooth Bud Organoids.; Figure S5: Histological evaluations of H&E-stained dental cell HMP constructs.
