# Supplementary material for: Self-Assembled Hydrogel Microparticle-Based Tooth-Germ Organoids
Source: Bioengineering (Basel). 2022 May 17;9(5):215. doi: 10.3390/bioengineering9050215 (PMC9137977; doi:10.3390/bioengineering9050215)
Supplement: Supplementary file 1 [file bioengineering-09-00215-s001.zip › bioengineering-1697098-supplementary.pdf]

bioengineering

MDPI

Article

Self-Assembled Hydrogel Microparticle-Based Tooth-Germ Organoids

Cemile Kilic Bektas [1,†], Weibo Zhang [2,†], Yong Mao [1], Xiaohuan Wu [1], Joachim Kohn [1] and Pamela C. Yelick [2,*]

[1] Department of Chemistry and Chemical Biology, Rutgers University, 123 Bevier Rd, Piscataway, NJ 08854, USA; cemilekilic87@gmail.com (C.K.B.); maoy@chem.rutgers.edu (Y.M.); xiaohuanwu2020@gmail.com (X.W.); kohn@dls.rutgers.edu (J.K.)
[2] Division of Craniofacial and Molecular Genetics, Department of Orthodontics, Tufts University School of Dental Medicine, 1 Kneeland Avenue, Boston, MA 02111, USA; weibo.zhang@tufts.edu (W.Z.)
* Correspondence: pamela.yelick@tufts.edu
† These authors contributed equally to this work.

Supplementary Data

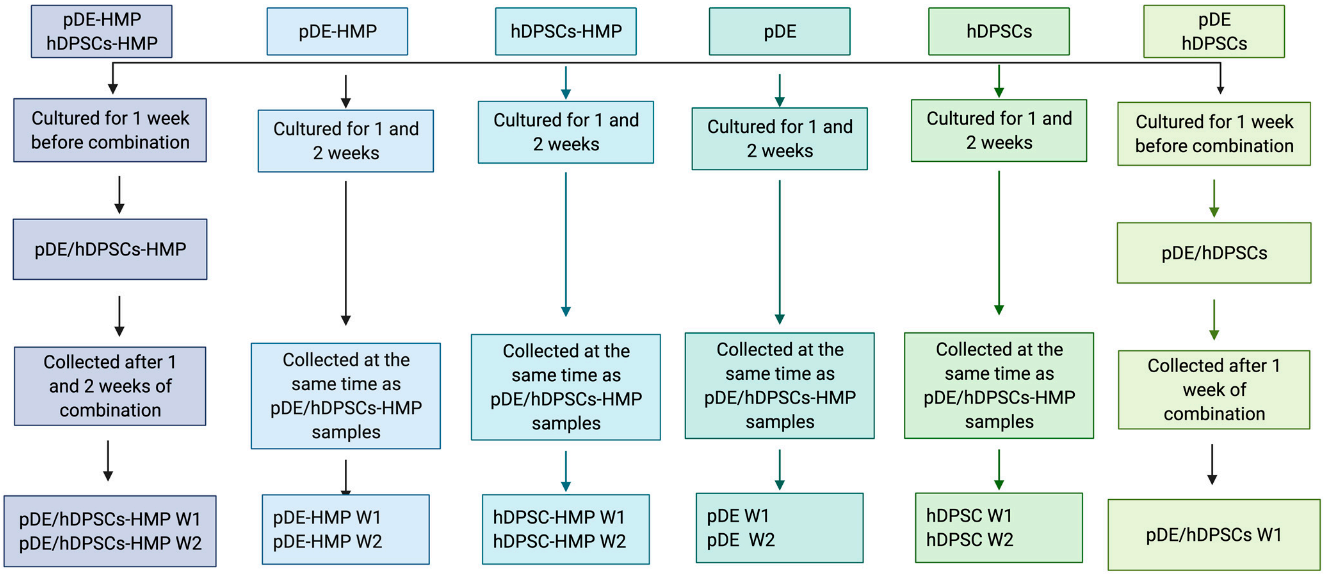

Figure S1. Flowchart of Tooth Bud Organoid Combination Testing.

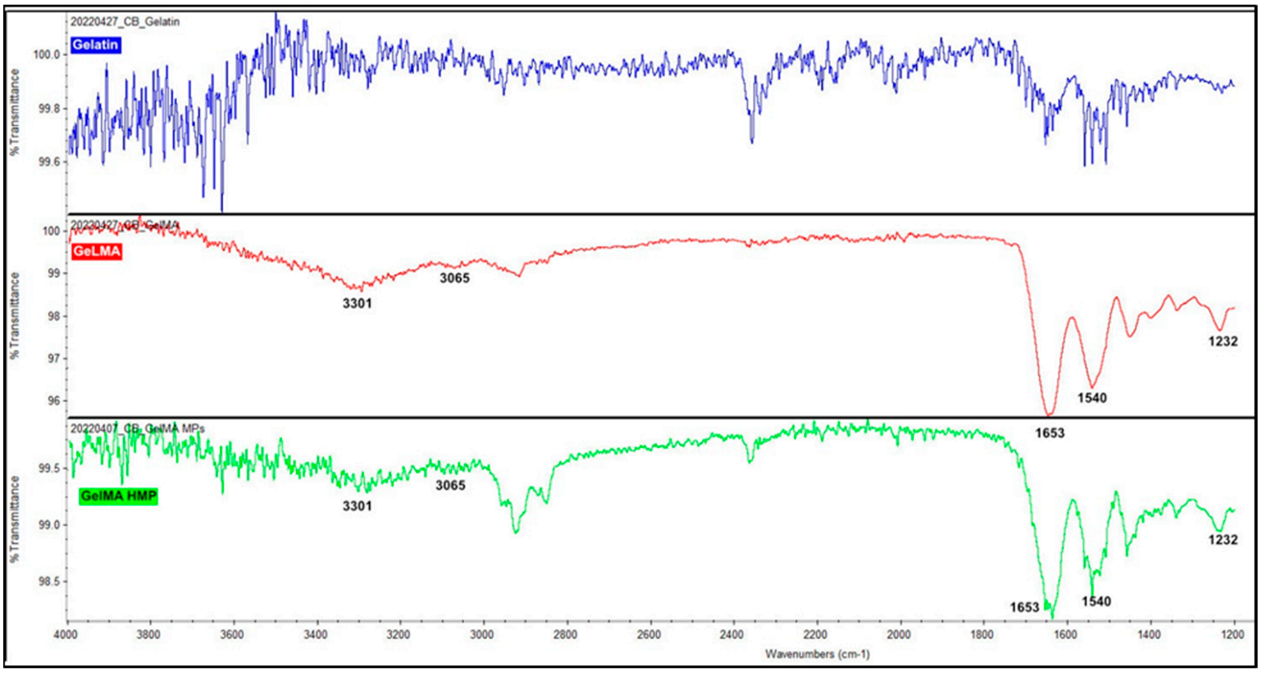

Figure S2. FTIR-ATR spectra. GelMA and GelMA hydrogel MPs exhibited the following peaks: the 3301 cm^{-1} peak is attributed to N-H stretching for Amide A band; the 3065 cm^{-1} peak is attributed to C-H stretching for Amide B band; the1653 cm^{-1} peak is attributed to C=O stretching for Amide I bond; the 1540 cm^{-1} peak is a mixture of N-H bending and C-N stretching for Amide II band; and the 1232 cm^{-1} peak belongs to N-H bending for Amide III band. The wave number of the Amide II band and presence of the Amide III band verify the secondary amide structure in GelMA and GelMA HMPs. These results confirm the successful fabrication of GelMA.

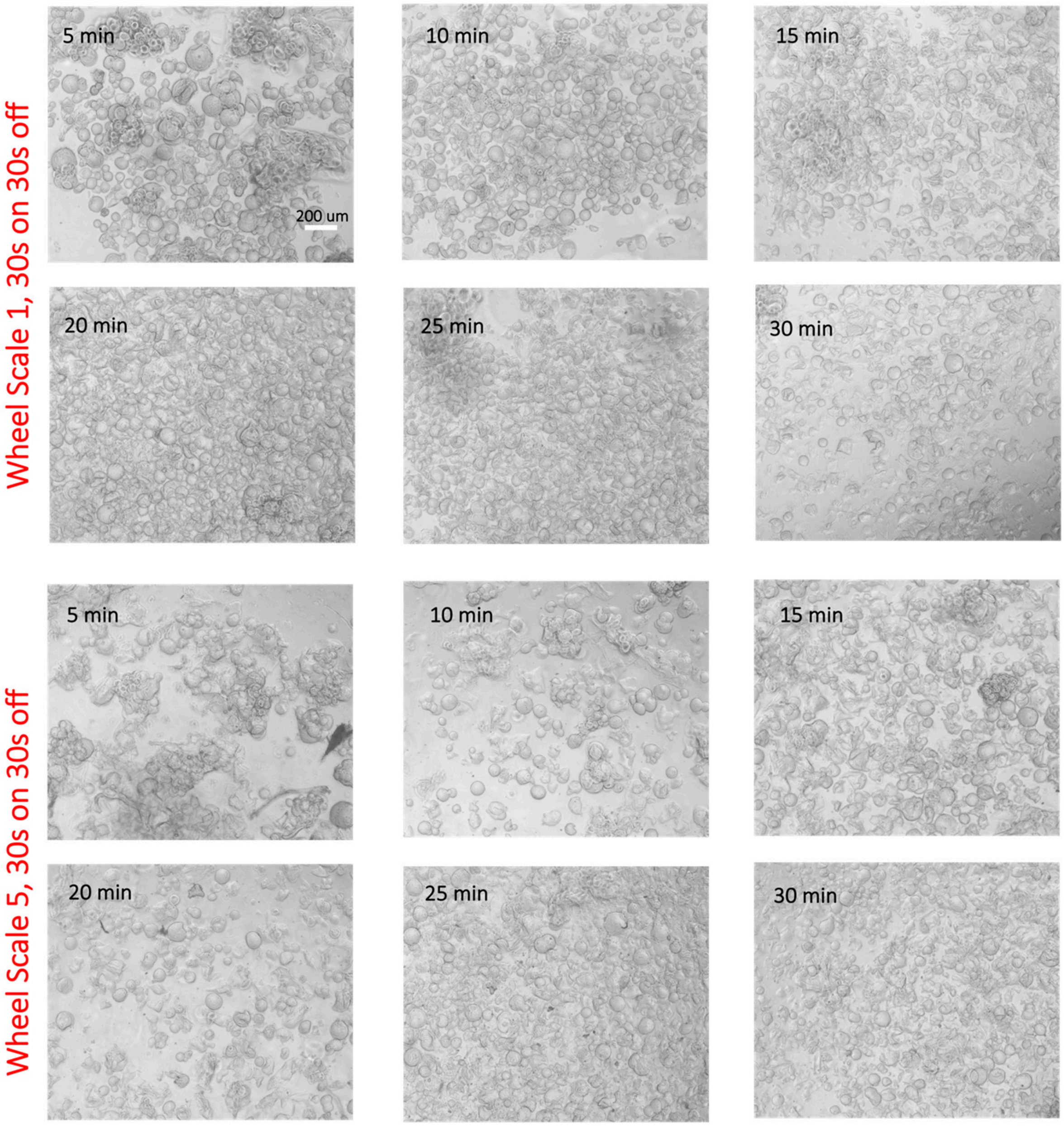

Figure S3. Effect of dispersion speed and duration on the microparticles. Increased fragmentation of HMPs was observed with increased dispersion speed and duration. Wheel scale 1 and 5 min dispersion yielded the best dissociation of the HMPs with negligible fragmentation.

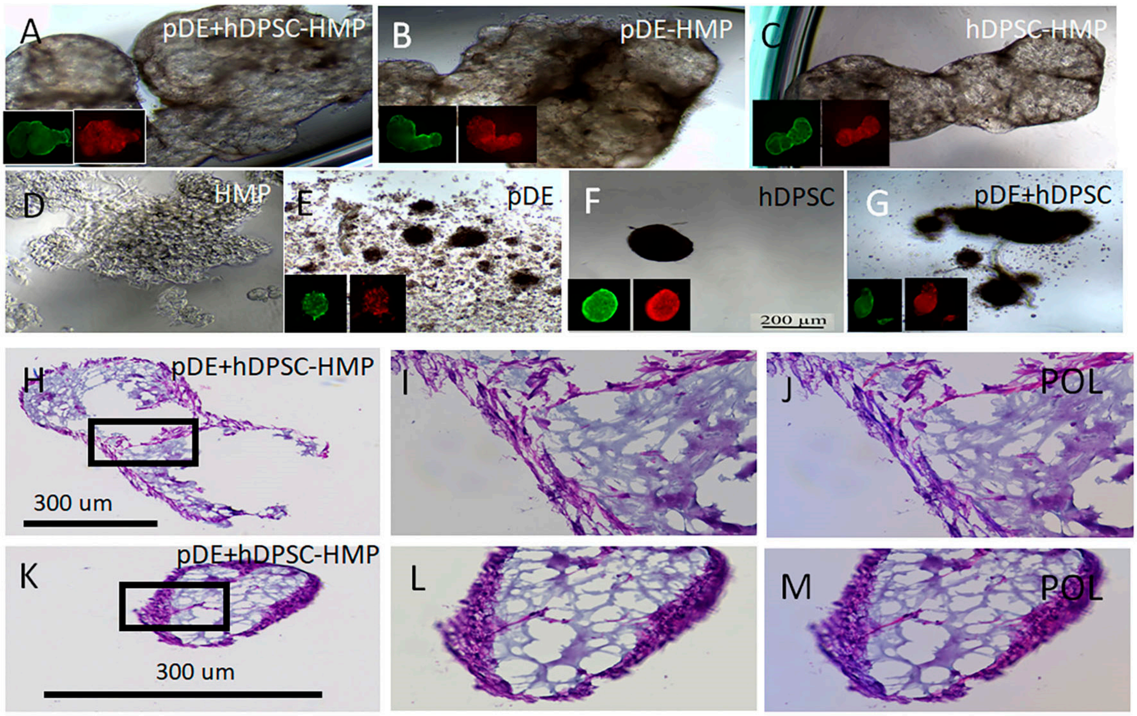

Figure S4. Two week in vitro cultured Tooth Bud Organoids. (A-G) Bright field images of dental cell and HMP organoids. Insets of live/dead assay showed higher cell viability for *in vitro* cultured cells with HMPs. Reduced cell viability (increased red) was observed in *in vitro* cultures without HMP. (H-M) Brightfield and polarized light (POL) microscopic images of H&E-stained recombinant dental cell HMP organoids shows high cellularity (dark purple) on HMPs (pale purple). Scale bar = 200 µm (A-G), (H-M) 300 µm.

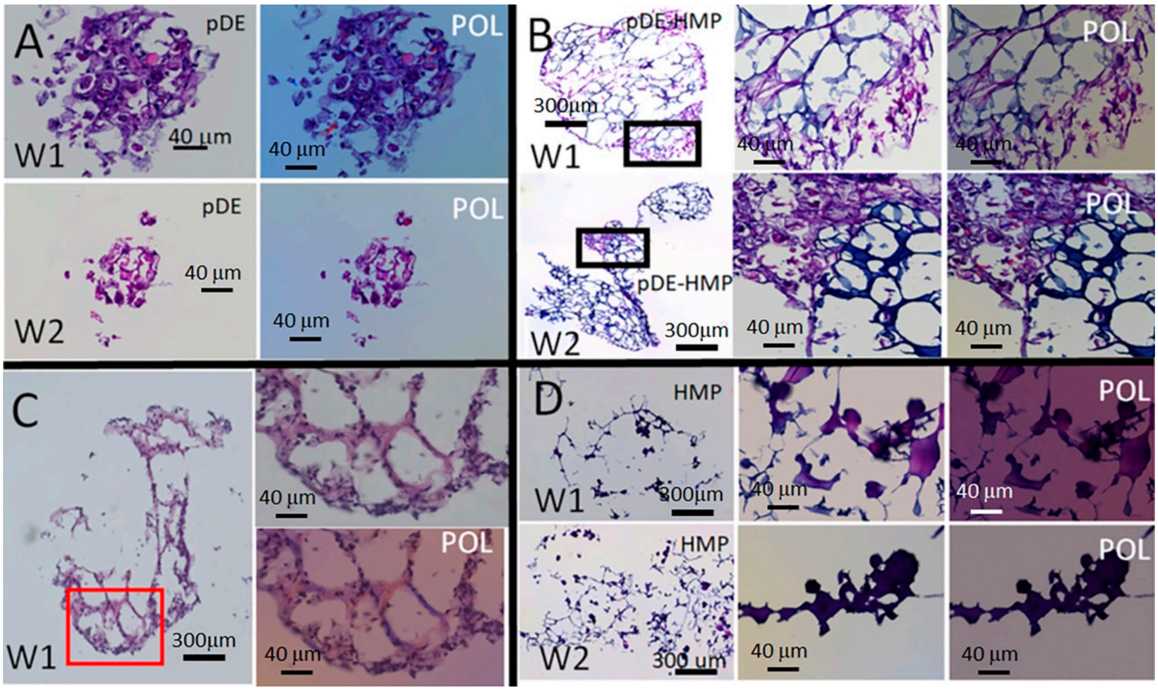

Figure S5. Histological evaluations of H&E-stained dental cell HMP constructs. A-B) pDE and C) hDPSCs showed typical cell morphologies in culture with or without HMPs. Individual cell types cultured with HMP did not exhibit polarized, differentiated phenotypes (A-C), in contrast to co-cultured cell HMP constructs which exhibited polarized cells (Fig. 4). D) HMP cultured alone stay dispersed. Scale bars are listed in each panel.
